# Rapid clonal identification of biallelic CRISPR/Cas9 knock-ins using SNEAK PEEC

**DOI:** 10.1038/s41598-023-28732-8

**Published:** 2023-01-31

**Authors:** Sameer Singh, Anoosha Banerjee, Arnaud Vanden Broeck, Sebastian Klinge

**Affiliations:** 1 Laboratory of Protein and Nucleic Acid Chemistry, The Rockefeller University, New York, NY 10065 USA; 2 Present Address: Institut für Medizinische Physik und Biophysik, Charité - Universitätsmedizin Berlin, corporate member of Freie Universität Berlin, Humboldt Universität zu Berlin, and Berlin Institute of Health, Berlin, Germany

**Keywords:** CRISPR-Cas9 genome editing, Genetic engineering

## Abstract

One of the challenges faced by current CRISPR/Cas9 editing strategies is the difficulty in rapidly selecting clonal populations of biallelically edited cells. Here we present Surface engiNeered fluorEscence Assisted Kit with Protein Epitope Enhanced Capture (SNEAK PEEC), a platform that combines human genome editing with cell-surface display, which enables the direct identification of biallelically edited clones with minimal screening.

## Introduction

CRISPR-Cas systems have allowed accurate editing of the human genome for applications in research and therapeutics^1–4^. However, current approaches suffer from low efficiencies, especially when inserting larger pieces of foreign DNA (knock-ins)^5–7^. As a result, CRISPR editing in human cells results predominantly in monoallelic knock-ins, wherein only a single gene copy (allele) is edited while the second may maintain its wildtype status or even undergo an undesired modification^8,9^. Hence, cells that undergo desired biallelic modifications represent a much smaller percentage of the entire edited population. This is especially of consequence in the field of structural biology, wherein affinity-tagging of endogenous proteins using CRISPR-CAS systems is often the only viable strategy to purify large, multicomponent complexes in their native, biologically active forms. Monoallelic editing or ectopic expression of tagged proteins can result in insufficient amounts of sample, impeding subsequent structural characterization of target complexes. Current selection strategies primarily utilize antibiotic selection or fluorescence assisted cell sorting (FACS) to identify biallelically edited cells^10–13^ . One drawback of antibiotic selection is that using multiple antibiotics can be harsh on cells and require additional screening of suitable selection conditions^10,13,14^,. Although intein-split antibiotic resistance genes can reduce the number of antibiotics required, they are insufficient to drive selection when expressed from endogenous promoters^10^. While current FACS based strategies are sensitive enough to detect fluorescence markers expressed from several native promoters, they are more conducive to selecting bulk polyclonal populations of mixtures of edited cells, followed by extensive genomic screening until a biallelically edited clone is identified^8,11,12^. This is arduous and resource intensive, leading to increased experimental turnaround times. There is therefore an urgent need to streamline the process of identifying biallelically edited cells.

## Results

**Figure 1:**
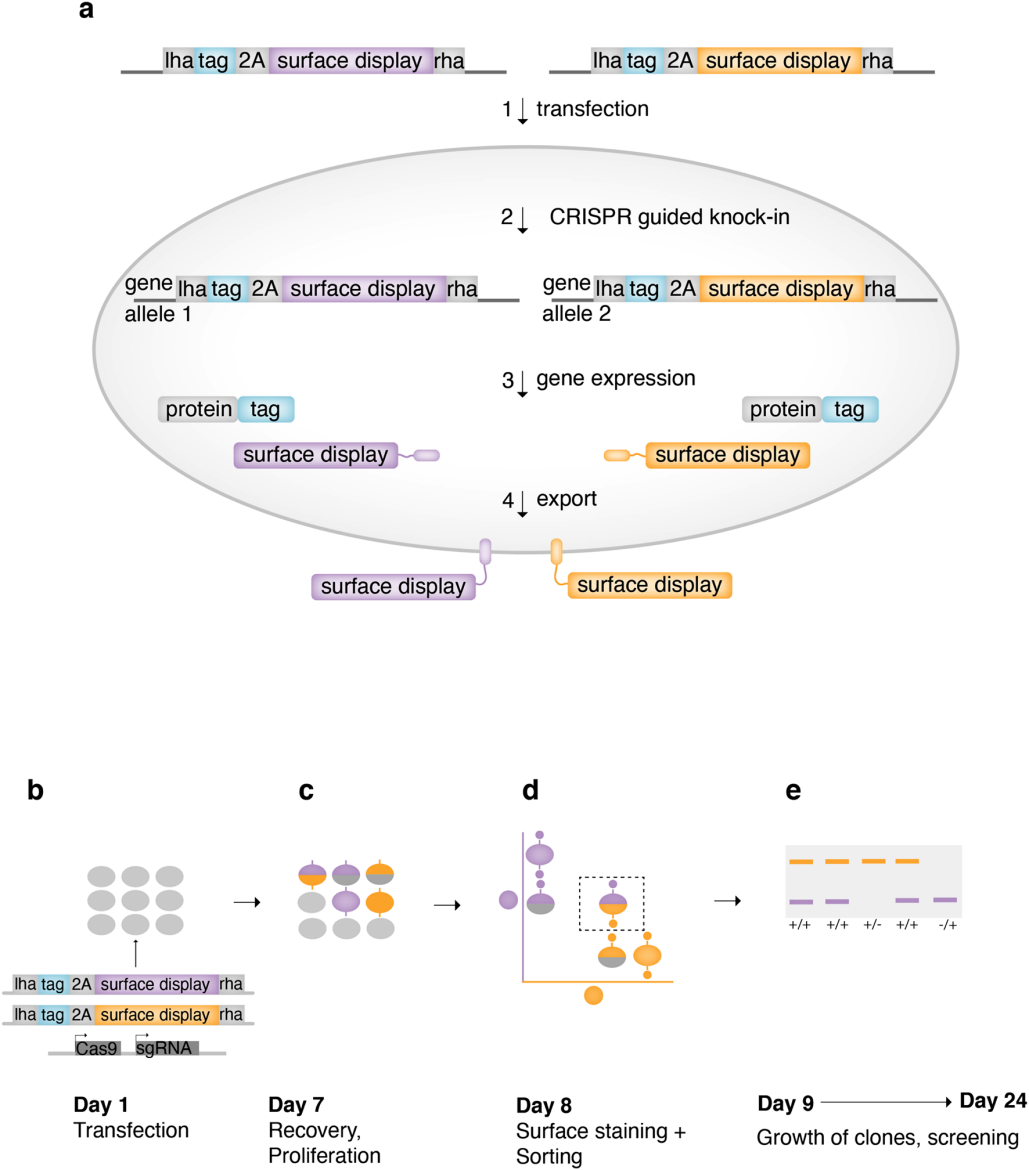
Selecting biallelic CRISPR knock-ins using SNEAK PEEC (**a**) Transfection of a cell with two repair templates and Cas9 and guide RNA enables knock-in at the 3’ end of both alleles of a target gene. Each repair template encodes an identical tag (blue), 2A self-cleaving peptide (grey) and unique cell-surface display (purple or yellow). Biallelically edited cells in which each allele has been targeted using a different repair template express the tagged protein as well as both unique cell-surface displays. Upon expression, each surface display is exported to the cell surface and displayed extracellularly. The 2A self-cleaving peptide ensures bicistronic expression of the tagged target protein and surface display (lha and rha; left and right homology arms). (**b**) Timeline for SNEAK PEEC. Transfection (Day 1) followed by cell recovery and proliferation (Day 7). Cells are surface stained and sorted for single cell clones expressing both surface displays (Day 8). Clones are expanded (Days 9–23), followed by PCR screening to confirm the genomic integration of both repair templates (Day 24), and hence biallelic editing.

For swift identification of biallelically edited cells, we developed an iterative genome editing platform called SNEAK PEEC (Surface engiNeered fluorEscence Assisted Kit with Protein Epitope Enhanced Capture), which combines CRISPR/Cas9 genome editing with cell-surface display (Fig. 1, Supplementary Fig. 3). While previous studies have employed surface tags to mainly identify bulk populations of CRISPR gene knockouts^11^, we have designed and implemented a strategy that allows the precise identification of clonal, biallelically edited knock-ins. The system utilizes two DNA repair templates, each encompassing an identical insert (e.g., a C-terminal tag) followed by a cell-surface display sequence encoding a unique protein epitope (Fig. 1a). Transfection of these repair templates with Cas9 and single guide RNA enables knock-in at the double strand break site located in the open reading frame (ORF) of the target gene. Thus, only in-frame knock-ins express a tagged protein and surface display. Biallelically edited cells in which each allele has been edited using a different repair template express the tagged protein from both alleles, as well as both surface displays (Fig. 1a). A viral 2A skipping peptide sequence ensures bicistronic expression of the tagged target protein and surface display^15^.

Transfection of human cells with the two DNA repair templates and a plasmid expressing Cas9 and single guide RNA can therefore result in six different outcomes of cells either containing no edited gene or different monoallelic ( − / +) or biallelic (+ / +) combinations (Fig. 1b, c). Of these outcomes only one includes both surface displays, which also represents a biallelically edited clone. Surface staining of cells with fluorescent antibodies (or nanobodies), each specific for only one of the surface display protein epitopes allows for the identification and isolation of cells expressing both surface displays from a much larger pool (Fig. 1d). Only a few clones need to be sorted for growth expansion and subsequent screening by PCR to identify biallelically edited clones (Fig. 1e).

A major advantage of SNEAK PEEC is that it pinpoints and picks out single cell clones among rare populations of biallelically edited cells, even when the overall Crispr knock-in efficiency is low (Supplementary Table 1). Additionally, it can be executed using a range of commonly available display epitopes (Supplementary Table 2). It thus allows for iterative editing at multiple loci in the same cell line, with each successive editing event utilizing a distinct display epitope pair. An added advantage of SNEAK PEEC is epitope recycling. Once a biallelically edited clone has been identified, both surface display encoding sequences can be excised from the genome using flanking, unidirectionally placed flippase recognition target (FRT) sites^16^. Excision is accomplished by transfecting edited cells with a plasmid that expresses the recombinase flippase, along with the fluorescent protein mtagBFP2. Single cell clones expressing the highest levels of mtagBFP2 (and hence the recombinase flippase) are selected using FACS, followed by PCR-based verification to ensure removal of both display epitopes (Supplementary Fig. 1). This feature yields 100% removal of both display epitopes, with the resulting clones retaining their functional biallelic edits. Furthermore, once recycled the epitope pair can be utilized for a new round of editing in the same cell line.

**Figure 2:**
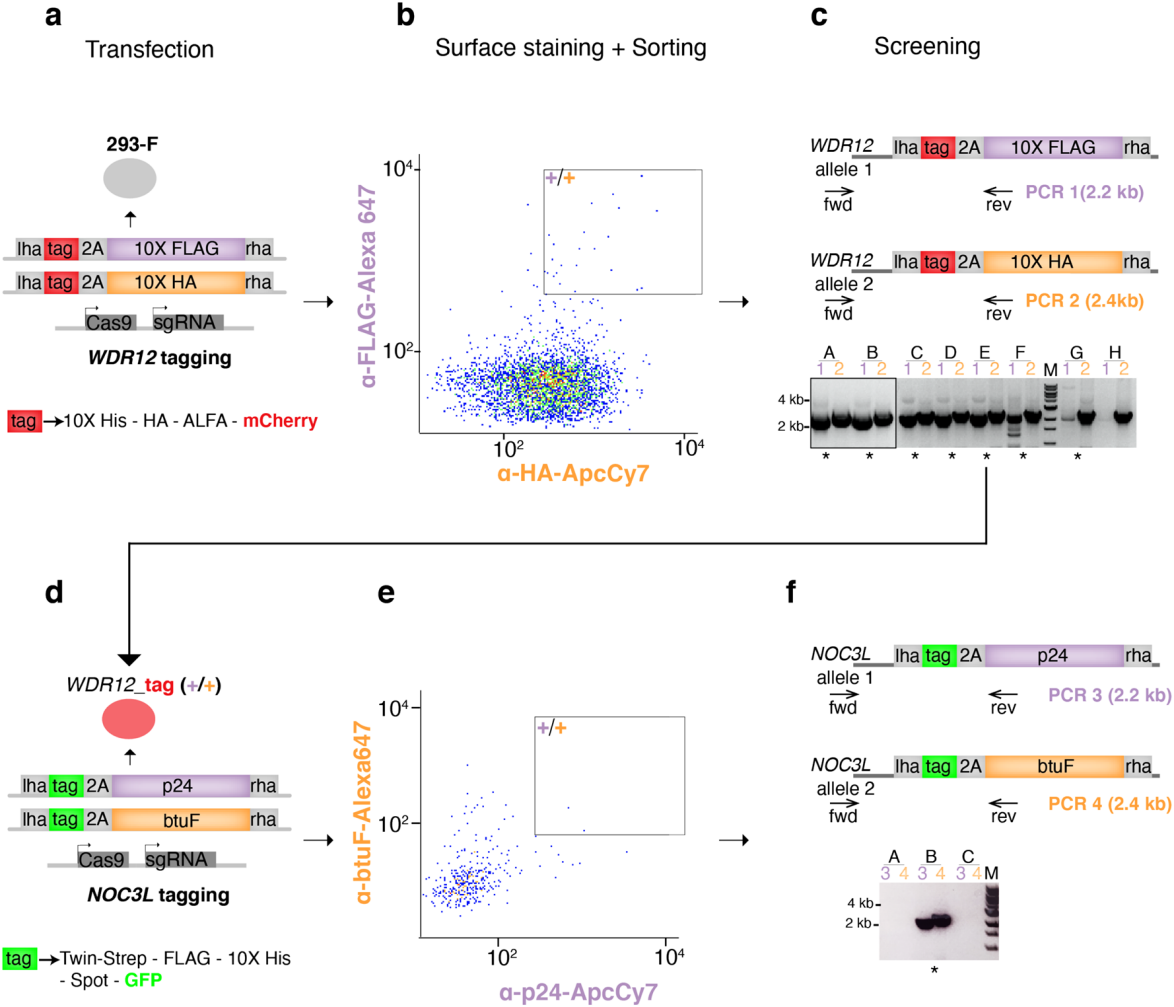
Iterative biallelic tagging using SNEAK PEEC. Biallelic tagging of the WDR12 gene using SNEAK PEEC (**a**–**c**). Transfection of 293-F cells with knock-in plasmids for biallelic C-terminal tagging (**a**). Surface staining of the transfected cell population with fluorescent antibodies targeting both surface displays (10X FLAG, 10X HA) (**b**). FACS sort of tagged mCherry positive single cell clones that also stain for both display targeting fluorescent antibodies (black box) (**b**). Genomic screening PCRs to identify biallelically edited clones (clones A-H) (**c**). Clones positive for both PCR products are indicated by asterisks. A single clone (clone E) was expanded for biallelic tagging of the NOC3L gene using a new set of knock-in plasmids (**d**). Surface staining of the transfected cell population with fluorescent nanobodies targeting both new surface displays (p24, btuF^23,24^). FACS sort of mCherry/GFP positive single cell clones that stain for both display targeting nanobodies (black box) (**e**). Genomic screening PCRs to identify biallelically edited clones (clones A-C) (**f**). Gel images shown in (**c**) and (**f**) have been cropped. Original gel images are shown in supplementary Fig. 4.

To illustrate the utility of SNEAK PEEC we performed the iterative, biallelic C-terminal tagging of two genes coding for nucleolar proteins, WDR12 and NOC3L, utilizing repair templates ranging from 2.4 to 2.9 kb in size (Fig. 2). SNEAK PEEC was used to first identify cells in which WDR12 was biallelically tagged with 87.5% efficiency (Fig. 2a–c). Next, one of these clones was subjected to a second round of editing to tag NOC3L, followed by selection of biallelically edited clones with an efficiency of 33% (Fig. 2d–f). Edited clones were imaged and showed the expected nucleolar localization of both WDR12 and NOC3L (Supplementary Fig. 2). SNEAK PEEC was thus used to successfully identify biallelically edited cells for facile and iterative genome editing events.

We further tested SNEAK PEEC in other cell types (Supplementary Table 4). However, low knock-in efficiencies (< 2%) in these cell lines highlight that SNEAK PEEC is currently optimized for HEK293-F cells and will require further optimization during future work in other cell lines. Furthermore, replacing plasmids with Cas9 ribonucleoproteins (RNPs) and viral vector^17,18^ will potentially aid in expanding the applicability of the technology to other cell types.

## Discussion and conclusion

Here we provide a tool that directly selects biallelically edited knock-ins using cell-surface displays, thereby eliminating the need for extensive clonal verification. By doing so SNEAK PEEC greatly reduces the considerable effort currently expended to obtain biallelically edited clones.

The ability to generate a clonal population with multiple edits is a growing area of scientific interest. We employed SNEAK PEEC to demonstrate this via the iterative biallelic tagging of two factors using distinct, non-overlapping surface display pairs. While the continued development of novel surface displays will permit more extensive editing in the future, the added feature of epitope recycling allows reuse of a display epitope for the selection of new editing targets. Overall, through continued/seamless biallelic tagging of human genes, SNEAK PEEC will aid in accelerating the structural characterization of elusive human protein complexes^19^. Overall, we envision this technology as a robust tool for the genetic editing of HEK293F cells, which are ubiquitously employed in biomedical research.

### Cell culture

Human kidney embryonic cells (293-F, ThermoFisher Scientific) were grown in Freestyle™ 293 Expression Medium (ThermoFisher Scientific, 12338026) supplemented with 2% heat inactivated FBS (ThermoFisher Scientific) and 1X Antibiotic–Antimycotic (ThermoFisher Scientific, 15240062), in an incubator at 37 °C and 8% CO_2_.

### Plasmid construction and transfections

All repair template plasmids contained left and right homology arms that covered a range of ≈ 600 bp in either direction of the Cas9 cut site. Homology arms separated by a multiple cloning site (MCS) were first cloned into pUC57 vectors (GenScript, Piscataway, NJ). The entire knock-in sequence was then cloned in using NotI/PacI restriction enzyme sites in the MCS. A plasmid expressing a single guide RNA (sgRNA) together with a high specificity *S. pyogenes* Cas9 variant (eSpCas9(1.1)) was a gift from Feng Zhang^20^ (Addgene plasmid # 71814). The 20-bp sgRNA target sequence was cloned into this vector using a pair of BbsI sites. The sgRNA sequences were selected using an online resource, crispor.telfor.net^21^. Guide RNAs with the highest specificity scores, and which targeted the last exon of the gene were selected for the experiments. Selected guide RNAs successfully edited their target DNA. Multiple guide RNAs were not tested for their editing efficiency.

The display removal plasmid expressed Flp recombinase as well as mTagBFP2, separated by a P2A sequence.

Transfections were carried out on cells grown in 24-well plates (Falcon, 353047). For SNEAK PEEC transfections each well received 1000 ng of total DNA, split in equimolar concentrations of each plasmid (Repair template 1, Repair template 2, Cas9 + sgRNA). For SNEAK PEEC display recycling transfections, each well received 500 ng of the display removal plasmid. All transfections in HEK293-F cells were carried out in 500 µl of Opti-MEM™ medium/well (ThermoFisher Scientific, 31985070) using the commercial transfection reagent Lipofectamine 2000 (ThermoFisher Scientific, 11668019). Nucleofections were carried out with the 4D-Nucleofector X Unit (Lonza, AAF-1003X) using the SE Cell Line 4D-Nucleofector X Kit S (Lonza, V4XC-1032) and the SF Cell Line 4D-Nucleofector X Kit S (Lonza, V4XC02032) for HeLa cells and HepG2 cells respectively. Transfections in HeLa cells were carried out using Lipofectamine 2000 (see above) as well as TransIT-HeLaMONSTER (Mirus, MIR2900). Cells were transfected at 70–90% confluency. After 14–16 h post transfection, cells were washed and resuspended in Freestyle™ 293 Expression Medium (ThermoFisher Scientific) supplemented with 2% heat inactivated FBS. Cells were allowed to recover, after which they were expanded to 6-well plates (VWR, 10062–892) for at least 7 days.

### Nanobody purification

*E.coli* BL21 RIL cells carrying a plasmid that encodes a nanobody directed against a cell surface epitope (Supplementary Table 2) were grown at 37 °C in 2x YT with 30 μg/ml kanamycin to OD_600_ of 2.0. Overexpression of the fusion protein was induced with 1 mM IPTG for 20 h at 28 °C. Cells were pelleted and resuspended in lysis buffer (20 mM HEPES, pH 7.7, 500 mM NaCl, 0.1% Triton) supplemented with 1X Pepstatin, 1X PMSF, 1X E-64, and DNase. Cells were subsequently lysed using a cell disrupter at 30 kPsi and the lysate was cleared by centrifugation at 40,000 × g for 30 min. The cleared lysate was supplemented with 30 mM imidazole and passed twice over a 5 mL IMAC His-Trap column. The column was washed with 30 mL of lysis buffer supplemented with 60 mM imidazole. The nanobody was eluted with 20 mL of lysis buffer supplemented with 300 mM imidazole. The 20 mL elution was then concentrated to 5 mL with a 10 kDa M.W cutoff concentrator (Pall) and injected over a HiLoad Superdex 75 gel filtration column that had been previously equilibrated in 1X PBS supplemented with 500 mM NaCl. Peak fractions were concentrated and stored at -80 °C until further use.

### Nanobody/antibody labeling

Labeling of nanobodies was carried out in-house using commercially available dyes. For labeling with Alexa 647 NHS Ester (ThermoFisher Scientific, A20006) nanobodies (Concentration, 0.5–1.2 mg/ml) were incubated with 7x molar excess of dye and placed on a shaker in the dark for 15 min, RT. The reaction was carried out in labeling buffer (100 mM NaHCO_3_ pH 7.8, 300 mM NaCl). A desalting column (GE Healthcare, 17085301) was used to separate excess free dye from dye labeled nanobodies, as well as perform a buffer exchange to quench the labeling reaction (Quenching buffer, 50 mM Tris/HCl pH 7.5, 300 mM NaCl, 250 mM sucrose). Apc-Cy7 labeling of nanobodies was carried out using a commercially available conjugation kit (AAT Bioquest, 2587). Briefly, nanobodies (Concentration, 0.5–1.2 mg/ml) were first pre-activated with Buccutite MTA by mixing on a shaker for 60 min, RT (8–10 µg Buccutite MTA/100 µg Nanobody). A desalting column was used to remove unreacted Buccutite MTA and exchange the buffer to 1X PBS. Reconstituted, pre-activated APC-Cy7 (premodified with Buccutite FOL) was directly added to the MTA-activated nanobody solution at the ratio of 130 µg APC-Cy7/100 µg MTA-activated Nanobody. The solution was rotated on a shaker for 60 min, RT thus enabling the reaction between the MTA-modified protein and FOL-modified APC-Cy7 that yields the APC-Cy7-Nanobody conjugate. The Anti-FLAG-Alexa647 antibody was purchased commercially (BioLegend, 637315). For the Anti-HA-Apc-Cy7 antibody, an Anti-HA-Biotin antibody (MilliporeSigma, 3F10) was mixed with a 5x molar excess of Streptavidin-APC-Cyanine7 (BioLegend, 405208) enabling the conjugation of a Biotin-streptavidin linked Anti-HA-APC-Cy7 fluorescent antibody. All labeled antibodies were stored at 4 °C and protected from light exposure.

### Flow cytometry

Cells were harvested from 6-well plates after a minimum of 7 days post transfection. Cells were first detached by gentle aspiration and washed 1x with 1X PBS, 0.1% BSA. They were then resuspended in 1X PBS, 0.1% BSA at a concentration of 1−10 X 10^6^ cells/ml. For SNEAK PEEC cell surface staining, requisite fluorescently labeled antibodies/nanobodies were added to 200 µl cell suspensions at a final concentration of 10 nM. Cells were labeled on ice in the dark for 30 min, washed 2x with 1X PBS, 0.1% BSA and resuspended in 200 µl of the same buffer. Samples were filtered prior to cell sorting to remove clumps (BD Falcon, 352235). DAPI was used to stain dead cells. For identification of biallelically tagged clones, sorting was used to first select cells expressing the fluorescent C-terminal tag, followed by selection of cells within this population that stained positive for both surface display epitopes. Cell sorting was carried out using a BD FACSAria cell sorter (BD Biosciences) using FACSDiva Software (BD Biosciences). For display recycling, cells were transfected with the display removal plasmid ≈ 48 h prior to FACS sorting. For identification of clones that had undergone display removal, sorting was used to select cells with the highest expression of the display removal plasmid. All necessary compensation was performed using single color controls before measurement and analysis of the data. FlowJo (FlowJo LLC) was used for analysis of the data.

### Genomic screening

Extraction of human genomic DNA was carried out using the QuickExtract DNA Extraction Solution (Lucigen, QE09050) as per the manufacturer’s protocol. 30 µl of extracted solution was used per screening PCR reaction (50 µl final volume). The following PCR primers were used in this study, WDR12 tagging, PCR1: fwd (CTCAGCCTCCCAGAGTGTTAGAATTAC), rev (GCCAGTACCCTTGTCATCGTCGTCT), PCR2: fwd (CTCAGCCTCCCAGAGTGTTAGAATTAC), rev (GAACCCCCGGTTCCGCTAG AGGTCGCATAAT). NOC3L tagging, PCR3: fwd (CTGAGGTGGATTTAGTTGGAT AATGTTGACATTACAATGG), rev (AGCGTTTTGTAGAACCGATCTACGTAA), PCR4: fwd (CTGAGGTGGATTTAGTTGGATAATGTTGACATTACAATGG), rev (GACTCCAC GGGGCCAACTGTCTCAAGG). Display recycling, Screening 1: fwd (GCAGGAGCGAACAATCTT TTTCAAGGA), rev (GAGAAACCTGCAGGTAAGATACATTGATGAGTTTGGACAA), Screening 2: fwd (CTGAGGTGGATTTAGTTGGATAATGTTGACATTACAATGG), rev (GAGAAAGGCGCGCCTTTATCCAAGTCAACCTTGTAGAGT).

### Imaging

Cells were first plated into individual wells of an 8 well chamber slide (Ibidi, 80,826) and grown until confluent. Attached cells were washed 1x with 1X PBS and fixed with 3.7% formaldehyde for 30 min, followed by another wash with 1X PBS. Samples were stored overnight in the dark at 4 °C. For DAPI staining cells were washed 3x with 1X PBS, followed by staining with 300 nM DAPI stain in the dark for 5 min. Excess DAPI was removed, and cells were washed 3x with 1X PBS.

### Equipment and settings

Imaging was carried out on a LSM880 Zeiss confocal microscope (Zeiss) using a LD LCI Plan-Apochromat 25x/0.8 Imm Korr DIC M27 lens, 1.8x  zoom using glycerol as an immersion medium. DAPI, GFP and mCherry were excited using 405, 488 and 561 nm laser lines, respectively. Images were adjusted for brightness/contrast using the software FIJI^22^.

### Ethical approval

No human subjects were involved in this study.

## Supplementary Information

Supplementary Information.

## Data Availability

The datasets generated during and/or analyzed during the current study are available from the corresponding author on reasonable request.
