# Supplementary material for: Rapid clonal identification of biallelic CRISPR/Cas9 knock-ins using SNEAK PEEC
Source: Sci Rep. 2023 Jan 31;13:1719. doi: 10.1038/s41598-023-28732-8 (PMC9889345; doi:10.1038/s41598-023-28732-8)
Supplement: Supplementary file 1 — Supplementary Information. [file 41598_2023_28732_MOESM1_ESM.pdf]

Rapid clonal identification of biallelic CRISPR/Cas9 knock-ins using SNEAK PEEC

Sameer Singh[1,†], Anoosha Banerjee[1], Arnaud Vanden Broeck[1], Sebastian Klinge[1,*]

[1] Laboratory of Protein and Nucleic Acid Chemistry, The Rockefeller University, New York, New York 10065, USA

[†]Present address: Institut für Medizinische Physik und Biophysik, Charité - Universitätsmedizin Berlin, corporate member of Freie Universität Berlin, Humboldt Universität zu Berlin, and Berlin Institute of Health, Berlin, Germany.

* email: klinge@rockefeller.edu

Supplementary Table 1: List of knock-in efficiencies based on fluorescence signal of transfected tag for factors biallelically tagged using SNEAK PEEC.

Biallelically tagged factor	In frame knock-in efficiency based on fluorescence signal of tag (Percentage of sorted population)
WDR12	24
NOC3L	8
UTP20	2.4
NOC4L	7.7
PES1	22.9

Supplementary Table 2: List of SNEAK PEEC display epitopes.

**Display Epitope**	**Length of display epitope (amino acids)**	**Source of Display Epitope**	**Display epitope detection molecule**	**PDB Code**
10X FLAG repeats	107	Recombinantly engineered [1]	fluorescently labeled antibody	-
10X HA repeats	180	Human influenza hemagglutinin (HA) surface glycoprotein from human influenza virus	fluorescently labeled antibody	-
btuF	245	Vitamin B12-binding protein from Escherichia coli	fluorescently labeled nanobody	5OVW [2]
p24	155	Capsid protein p24 from Human immunodeficiency virus 1	fluorescently labeled nanobody	5O2U [3]

porM	148	T9SS component cytoplasmic membrane protein PorM from Porphyromonas gingivalis	fluorescently labeled nanobody	6EY0 [4]
STAS	93	Sulphate transporter from Deinococcus geothermalis DSM 11300	fluorescently labeled nanobody	5DA0 [5]

Supplementary Table 3: Single guide gRNA (sgRNA) target sequences.

Target gene	sgRNA target sequence (5' – 3')	PAM (NGG)
WDR12	ACCTACCACTTCCCATGTTG	GGG
NOC3L	AGTTGCTACTGAATCGCCTC	TGG

Supplementary Table 4: Testing SNEAK PEEC in other cell types

Cell Type	Gene target	Transfection reagent/method	Result
HeLa	*Wdr12*	TransIT-HeLaMONSTER	Low knock-in efficiency (1.6%). No biallelically edited clones detected via FACS
		FuGene	Knock-in failed
		Lipofection-(Lipofectamine 3000)	Low knock-in efficiency (0.8%) No biallelically edited clones detected via FACS
		Reverse Lipofection-(Lipofectamine 3000)	Knock-in failed
		Nucleofection	Knock-in failed
HepG2	*WDR12*	Nucleofection	Low knock-in efficiency (0.5%). No biallelically edited detected

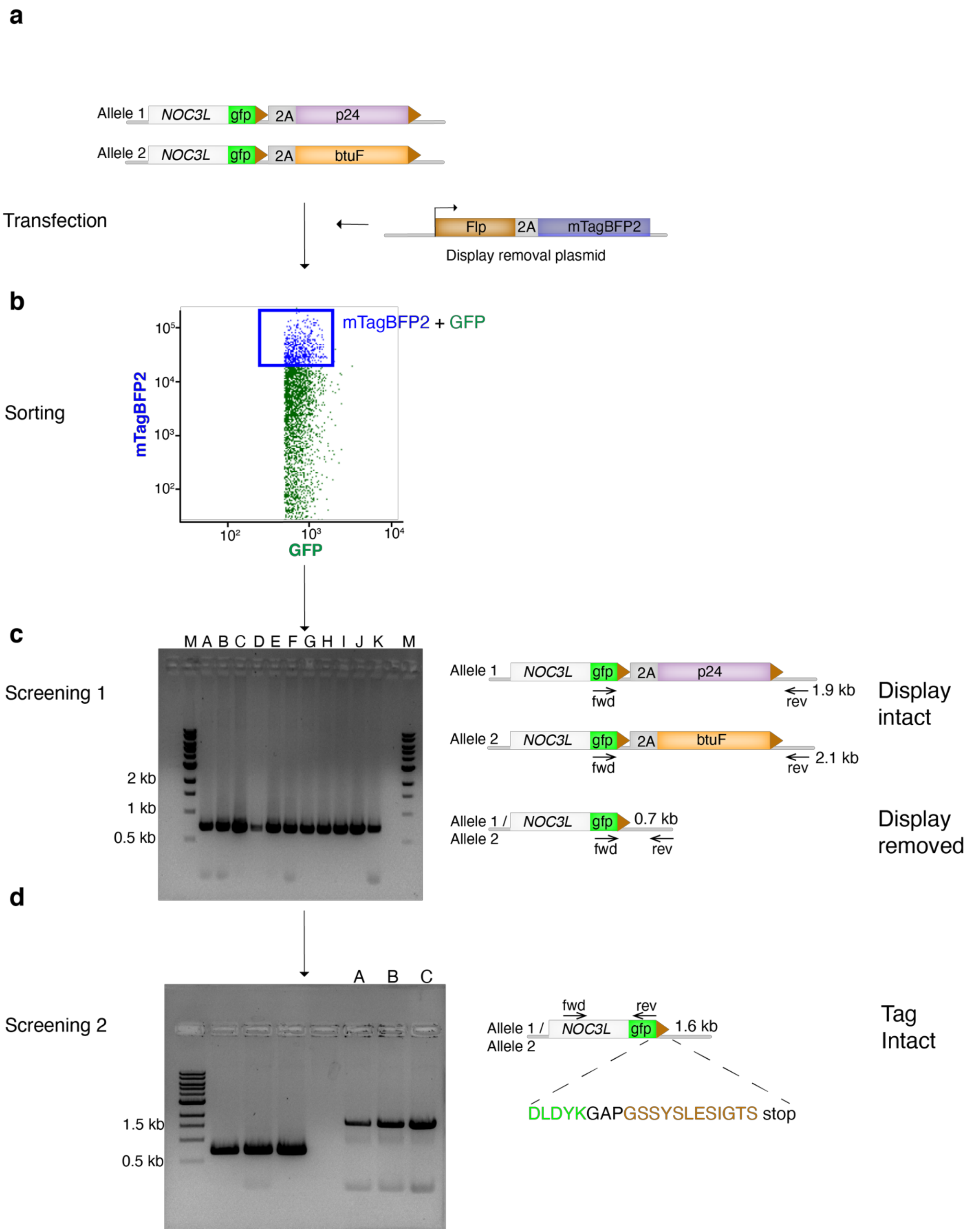
a
Allele 1 NOC3L gfp 2A p24
Allele 2 NOC3L gfp 2A btuF
Transfection
Flp 2A mTagBFP2
Display removal plasmid
b
Sorting
mTagBFP2 + GFP
mTagBFP2
GFP
c
Screening 1
M A B C D E F G H I J K M
2 kb
1 kb
0.5 kb
Allele 1 NOC3L gfp 2A p24
fwd rev 1.9 kb
Allele 2 NOC3L gfp 2A btuF
fwd rev 2.1 kb
Display intact
Allele 1 / Allele 2 NOC3L gfp 0.7 kb
fwd rev
Display removed
d
Screening 2
A B C
1.5 kb
0.5 kb
fwd rev
Allele 1 / Allele 2 NOC3L gfp 1.6 kb
Tag Intact
DLDYKGAPGSSYSLESIGTS stop

Supplementary Figure 1. Recycling of SNEAK PEEC display epitopes. Transfection of display removal plasmid (Flp-2A-mTagBFP2) targeting unidirectional FRT sites (brown arrowheads) present in both edited *NOC3L* alleles **(a)**. FACS sorting to select single cell clones expressing the highest levels of mTagBFP2 and GFP (blue box) **(b)**. First round of genomic screening PCRs to identify clones (clones A-K) that have undergone display removal **(c)**. Allele architecture and expected sizes of amplified DNA are shown on the right. M: Molecular weight marker, fwd: Forward PCR primer, rev: Reverse PCR primer. Three clones (clones A-C) from **(c)** were selected for further genomic screening to confirm preservation of the tag after display removal **(d)**. The C-terminal amino acid sequence retained after display removal is listed within dashed lines (green: GFP C-terminus, brown: single FRT site, stop: stop codon).

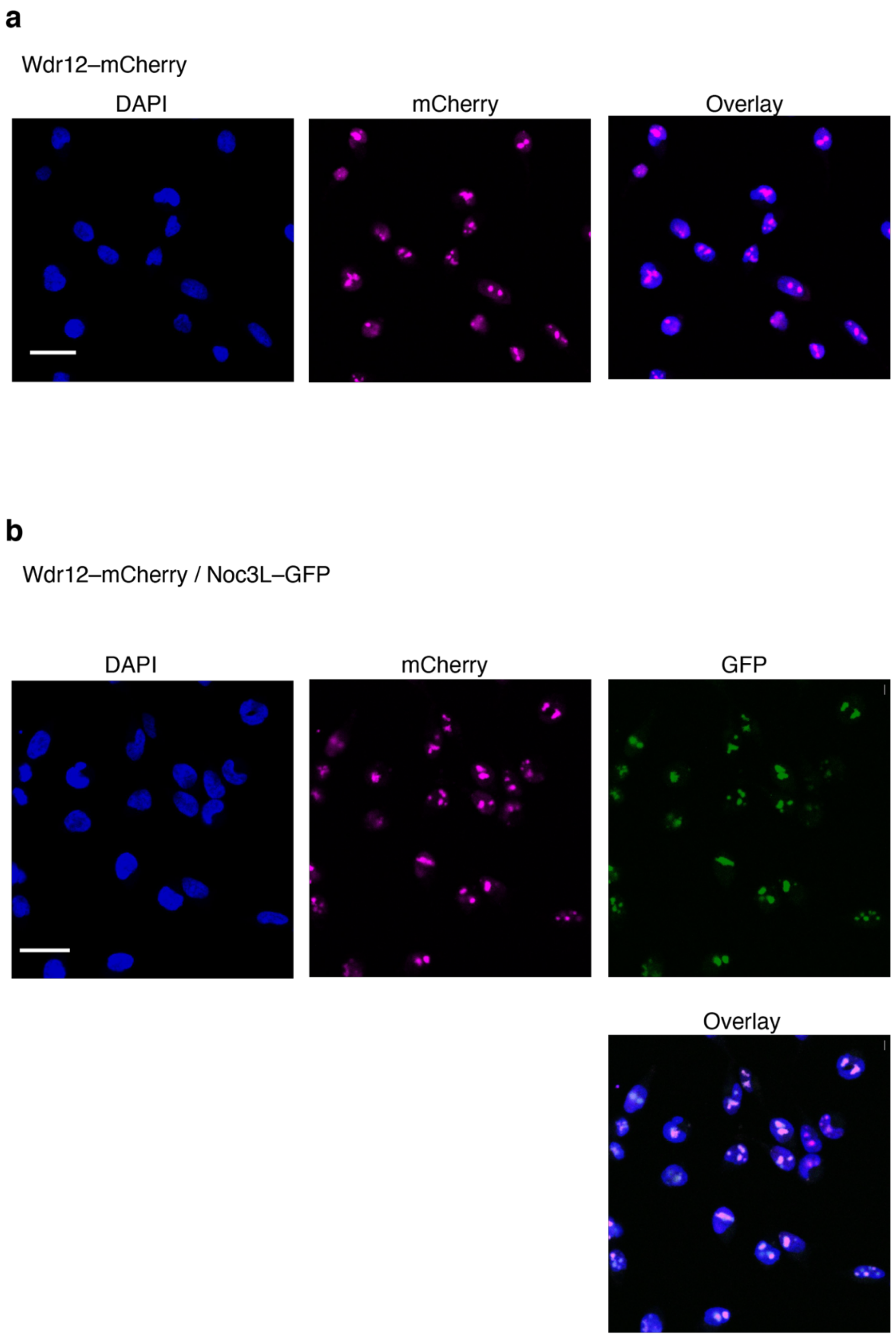

Supplementary Figure 2. Confocal imaging showing the results of iterative biallelic tagging using SNEAK PEEC. Clonal population showing nucleolar localization of mcherry tagged WDR12 **(a)** after the first round of editing. A second round of editing on this clone

showing nucleolar localization of mcherry tagged WDR12 as well as GFP tagged NOC3L **(b)**. (White bar: 30 *μ*m).

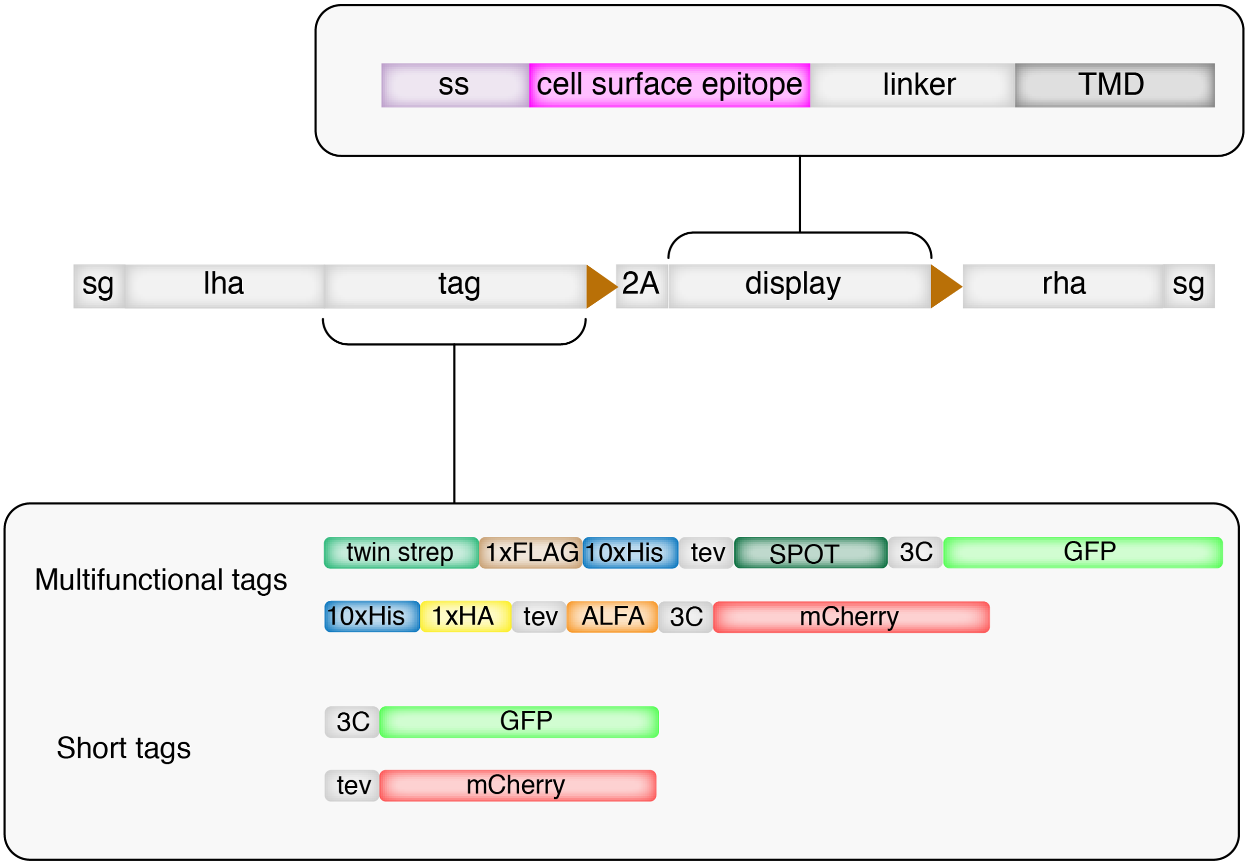

Supplementary Figure 3. Architecture of a SNEAK PEEC repair template. sg: sgRNA target sequence, lha: left homology arm, tag: C-terminal tag, 2A: self-cleaving peptide sequence, display: cell-surface display sequence, rha: right homology arm, sg: sgRNA target sequence. Unidirectional recombinase target sequences are displayed as brown arrowheads. The range of current available tags is shown within the box below the tag panel. Makeup of the display sequence is shown within the box above the display panel. ss: secretion signal, cell surface epitope, linker, TMD: transmembrane domain.

a

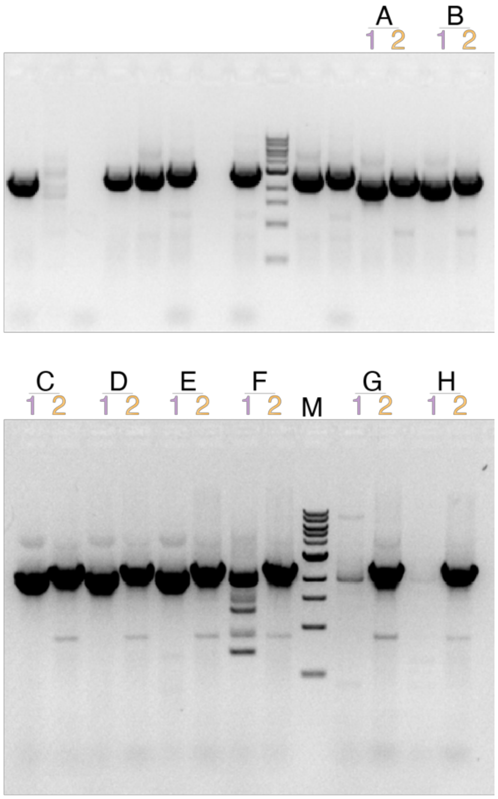

b

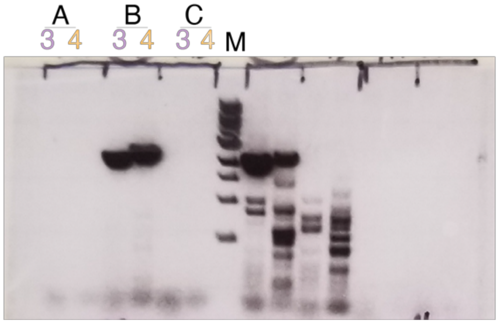

Supplementary Figure 4. Original agarose gels

Full gel pictures showing the results of the genomic screening PCRs to identify biallelically edited (a) *Wdr12* clones (clones A-H) and (b) *Noc3L* clones (A-C).

References

1. Hopp, T. P. *et al.* A short polypeptide marker sequence useful for recombinant protein identification and purification. *Bio/Technology* (1988) doi:10.1038/nbt1088-1204.
2. Mireku, S. A., Sauer, M. M., Glockshuber, R. & Locher, K. P. Structural basis of nanobody-mediated blocking of BtuF, the cognate substrate-binding protein of the Escherichia coli vitamin B12 transporter BtuCD. *Sci. Rep.* (2017) doi:10.1038/s41598-017-14512-8.
3. Gray, E. R. *et al.* Unravelling the Molecular Basis of High Affinity Nanobodies against HIV p24: In Vitro Functional, Structural, and in Silico Insights. *ACS Infect. Dis.* (2017) doi:10.1021/acsinfecdis.6b00189.
4. Leone, P. *et al.* Type IX secretion system PorM and gliding machinery GldM form arches spanning the periplasmic space. *Nat. Commun.* (2018) doi:10.1038/s41467-017-02784-7.
5. Geertsma, E. R. *et al.* Structure of a prokaryotic fumarate transporter reveals the architecture of the SLC26 family. *Nat. Struct. Mol. Biol.* (2015) doi:10.1038/nsmb.3091.
